# Current Trends and Future Opportunities of AI-Based Analysis in Mesenchymal Stem Cell Imaging: A Scoping Review

**DOI:** 10.3390/jimaging11100371

**Published:** 2025-10-18

**Authors:** Maksim Solopov, Elizaveta Chechekhina, Viktor Turchin, Andrey Popandopulo, Dmitry Filimonov, Anzhelika Burtseva, Roman Ishchenko

**Affiliations:** 1 V.K. Gusak Institute of Emergency and Reconstructive Surgery, 283045 Donetsk, Russia; 2 Medical Research and Educational Institute, Lomonosov Moscow State University, 119234 Moscow, Russia

**Keywords:** mesenchymal stem cells, deep learning, machine learning, artificial intelligence, imaging

## Abstract

This scoping review explores the application of artificial intelligence (AI) methods for analyzing mesenchymal stem cells (MSCs) images. The aim of this study was to identify key areas where AI-based image processing techniques are utilized for MSCs analysis, assess their effectiveness, and highlight existing challenges. A total of 25 studies published between 2014 and 2024 were selected from six databases (PubMed, Dimensions, Scopus, Google Scholar, eLibrary, and Cochrane) for this review. The findings demonstrate that machine learning algorithms outperform traditional methods in terms of accuracy (up to 97.5%), processing speed and noninvasive capabilities. Among AI methods, convolutional neural networks (CNNs) are the most widely employed, accounting for 64% of the studies reviewed. The primary applications of AI in MSCs image analysis include cell classification (20%), segmentation and counting (20%), differentiation assessment (32%), senescence analysis (12%), and other tasks (16%). The advantages of AI methods include automation of image analysis, elimination of subjective biases, and dynamic monitoring of live cells without the need for fixation and staining. However, significant challenges persist, such as the high heterogeneity of the MSCs population, the absence of standardized protocols for AI implementation, and limited availability of annotated datasets. To advance this field, future efforts should focus on developing interpretable and multimodal AI models, creating standardized validation frameworks and open-access datasets, and establishing clear regulatory pathways for clinical translation. Addressing these challenges is crucial for accelerating the adoption of AI in MSCs biomanufacturing and enhancing the efficacy of cell therapies.

## 1. Introduction

Mesenchymal stem cells (MSCs) are multipotent cells capable of differentiating into osteoblasts, chondrocytes, and adipocytes, making them a crucial focus in regenerative medicine research [1]. Their high proliferative activity, immunomodulatory properties, and ability to maintain tissue homeostasis render them promising for anticancer therapy, as well as the treatment of inflammatory and fibrotic diseases [2]. The ability of MSCs to migrate to damaged tissues ensures targeted therapeutic action, which is particularly valuable for cell therapy. Recent studies have actively explored the use of MSCs in nerve tissue repair [3], musculoskeletal tissue regeneration [4,5], immunomodulation in autoimmune diseases [6], the treatment of ischemic conditions [7] and the development of anticancer strategies. These strategies include the use of MSCs as carriers for anticancer drugs and genes, employing exosomes for cell-free therapy, inhibiting Wnt/β-catenin and PI3K/AKT/mTOR signaling pathways, as well as immunomodulation and targeted action on tumors [8]. Numerous clinical trials are currently underway to assess the efficacy and safety of MSCs in treating various pathologies [9,10,11,12,13]. However, the complexity of MSCs interaction with the microenvironment, including the influence of cytokines, growth factors, and the cellular milieu, necessitates further research to optimize their therapeutic applications.

In vitro visualization under laboratory culturing conditions is essential for the detailed characterization of MSCs and quality control of cell cultures, which is critical for standardization and ensuring safety in clinical applications. Traditional visualization methods, such as light microscopy, fluorescence microscopy, and confocal microscopy, are widely used to analyze cell viability, morphology, structure, differentiation, and interaction with the surrounding environment. Modern imaging techniques, such as magnetic particle imaging (MPI) and positron emission tomography (PET), provide additional insights into the location and viability of MSCs in living organisms, crucial for assessing their therapeutic potential [14]. Techniques like three-dimensional visualization and fluorescence microscopy with fluorescence lifetime imaging (FLIM) enable detailed studies of cellular functions and structural changes, including MSCs interactions with the microenvironment and evaluation of cell viability and migration [15]. MRI with mannosyl labeling allows for cell tracking without exogenous markers, minimizing potential data distortion [16]. Despite their significant advantages, these methods have limitations, such as the need for specialized equipment, operator expertise, potential alterations in cell properties due to labeling, and challenges in data interpretation, all of which require careful consideration.

Manual image analysis of MSCs faces several limitations, including subjective assessments and the lack of standardized evaluation criteria [17]. It negatively impacts the reproducibility of experiments and data reliability [18]. This method requires significant time investment and limits the processing of large datasets, making it inefficient for large-scale studies or real time monitoring [19,20]. Additionally, manual analysis may not detect subtle morphological changes, limiting its sensitivity [18,21]. These shortcomings render manual analysis unsuitable for tasks requiring high precision and throughput, highlighting the need for automated analysis methods, including those based on artificial intelligence (AI).

AI methods utilize mathematical models to semiautomatically extract, analyze, and interpret image data without direct human involvement. Artificial neural networks are commonly used. These algorithms are trained on large datasets to identify complex patterns and relationships. During training, model parameters are adjusted based on prediction errors, allowing algorithms to improve accuracy over time. AI methods automate processes such as cell segmentation and detection, classification of cell states (e.g., normal or senescent cells), real-time tracking of cellular changes, and quantitative assessment of cell viability and differentiation parameters [22,23,24]. The key advantages of AI include high data processing speed and scalability, enabling adaptation to various tasks. Additionally, AI algorithms can identify hidden patterns in data that might be overlooked in manual analysis, which is particularly important for heterogeneous cell populations. AI ensures standardization of analysis, eliminating subjective variability, and can predict the dynamics of cellular processes, crucial for developing new therapeutic strategies and monitoring cell culture conditions in real time [25].

AI methods for MSCs visualization hold significant potential; however, the existing research is fragmented and lacks systematic organization. The diversity of approaches used creates challenges in evaluating their effectiveness and comparing results. The lack of a unified knowledge base limits the possibilities for standardizing methods, improving reproducibility, and ensuring objectivity.

While previous reviews, including that by Issa et al. [24], have provided valuable overviews of AI applications in stem cell imaging, our work contributes uniquely in three key aspects. First, we focus exclusively on mesenchymal stem cells (MSCs), enabling a deeper analysis that reflects their distinct biological properties. Second, and most importantly, we introduce a novel dual-axis classification framework: studies are categorized both by AI algorithm type and by the biological task addressed, such as MSC senescence and differentiation. This approach uncovers trends that are technological, scientific, and application-oriented, distinguishing our review from earlier efforts.

Therefore, this scoping review not only consolidates disparate studies but integrates them into a new analytical framework, highlights key achievements and challenges, and offers insights valuable for both researchers and practitioners in cell technologies and regenerative medicine.

## 2. Materials and Methods

### 2.1. Protocol

Prior to initiating the review, a comprehensive research protocol was developed. This protocol detailed the objectives, methods, and inclusion criteria, following the methodology for writing scoping reviews as outlined by the Joanna Briggs Institute (JBI) [26] and adhering to the key principles of PRISMA-ScR [27]. The review protocol is available upon request from the corresponding author.

### 2.2. Research Question

The main research question was formulated using the population, concept, and context (PCC) framework [26]:

- Population: MSCs of any type, from either animal or human sources;
- Concept: application of AI methods for image processing;
- Context: analysis of cell culture images.

Based on these components, the primary research question was defined as: “For what purposes are AI methods used in the processing of MSCs images?”

### 2.3. Search Strategy

In October 2024, a comprehensive search was conducted across six electronic databases (PubMed, Dimensions, Scopus, Google Scholar, eLibrary, and Cochrane) to identify relevant studies addressing the research question. Search queries were adapted to match the syntax of each database (Table S1). The search queries included keywords, their variations, and abbreviations: “artificial intelligence”, “convolutional neural network”, “deep learning”, “machine learning”, “transfer learning”, “mesenchymal stem cells”, “authentication”, “classification”, “detection”, “identification”, “image processing”, “imaging”, “prediction”, and “screening”.

Due to the limitations of Google Scholar in handling complex search queries, the search strategy utilized the string: imaging AI, OR artificial OR intelligence, OR convolutional OR neural OR network, OR deep OR learning, OR machine OR learning, OR transfer OR learning “mesenchymal stem cell*”. The first 200 records returned by this query were assessed for relevance. Additionally, a manual citation search was performed by reviewing the reference lists of included studies to identify potential works addressing the research question.

Search results were imported and saved into a collection using Zotero 7.0 (Corporation for Digital Scholarship, Vienna, VA, USA). Duplicates were removed from the collection using the Rayyan web service for systematic reviews [28]. S.M. manually verified the removal of duplicates.

### 2.4. Study Selection and Data Extraction

Inclusion and exclusion criteria for studies were developed based on the PCC mnemonic [26] and presented in Table 1.

To ensure a consistent and standardized application of these criteria, the review team (S.M., C.E., and T.V.) first conducted a calibration exercise on a sample of articles to align their interpretations. The selection process was conducted in two distinct stages. In the first, two reviewers (S.M. and C.E.) independently screened the titles and abstracts of all retrieved records. In the second, the same reviewers independently assessed the full texts of potentially eligible articles for final inclusion. Disagreements at either stage were resolved through discussion. If consensus could not be reached, a senior reviewer (T.V.) was consulted to make the final decision. The Rayyan web service [28] was utilized for collaborative management of study metadata.

Data extraction was performed by one reviewer (S.M.) using a standardized form and subsequently verified by a second reviewer (C.E.). For analysis, the following data were extracted from each study: authors, year of publication, country of the study, study objective, cell type and origin, AI algorithm, dataset description, and research outcomes.

## 3. Results

### 3.1. Search Results

**Figure 1:**
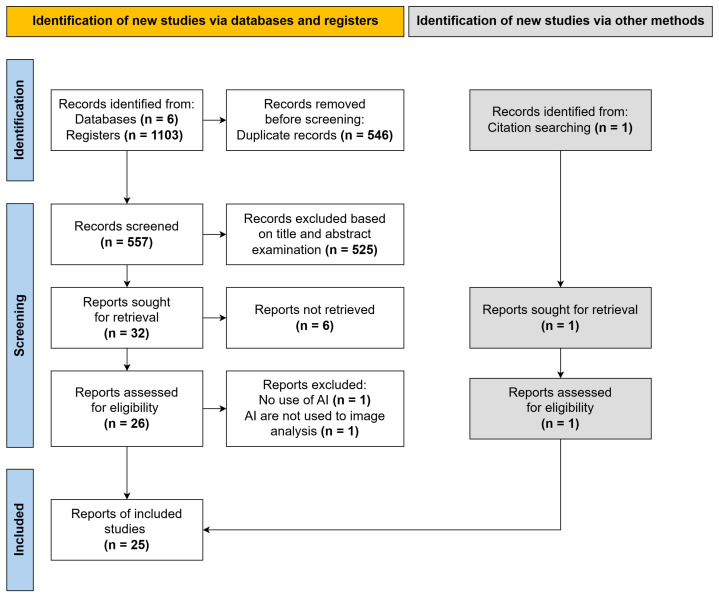
PRISMA diagram. This diagram illustrates the comprehensive methodology of the scoping review, detailing the number of articles identified at each stage of the database search, the number of duplicates excluded, and the count of articles that underwent title and abstract screening, full text analysis, and were ultimately included in the review.

A comprehensive search across six electronic databases yielded a total of 1103 articles. After removing duplicates, 557 articles remained for title and abstract screening against the inclusion and exclusion criteria. Following this initial screening, 32 articles were selected for full text analysis. However, six articles were excluded due to the unavailability of the full text. Two more articles were excluded after full text review, as one did not utilize AI methods and the other applied AI for purposes unrelated to image analysis. An additional article was included through manual citation searching. Ultimately, 25 studies were incorporated into this scoping review (Figure 1). The inter-rater agreement, calculated using the kappa statistic, was K = 0.886, indicating substantial agreement among the reviewers.

### 3.2. Characteristics of Included Studies

**Figure 2:**
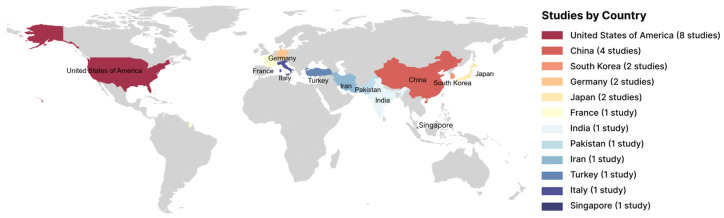
Geographical distribution of included studies. The map illustrates the geographical origin of the research groups included in the review, highlighting countries that have made the most significant contributions to the study of artificial intelligence (AI) applications in mesenchymal stem cells (MSCs) image analysis. The distribution reflects the dominant positions held by the USA (32% of studies) and China (16%).

The extracted data is summarized in Table 2. Geographic distribution (Figure 2) reveals that research groups from the United States and China hold dominant positions; this likely indicates the presence of major government programs and substantial private investment in the fields of regenerative medicine and AI within these countries. Research teams from the United States contributed the largest share, with 8 out of 25 studies (32%) [20,29,30,31,32,33,34,35]. Chinese researchers published 4 studies (16%) [36,37,38,39]. Teams from South Korea [40,41], Germany [42,43], and Japan [44,45] each contributed 2 studies (8%), while France [46], India [21], Pakistan [47], Iran [48], Turkey [49], Italy [50], and Singapore [51] each published 1 study (4%).

**Figure 3:**
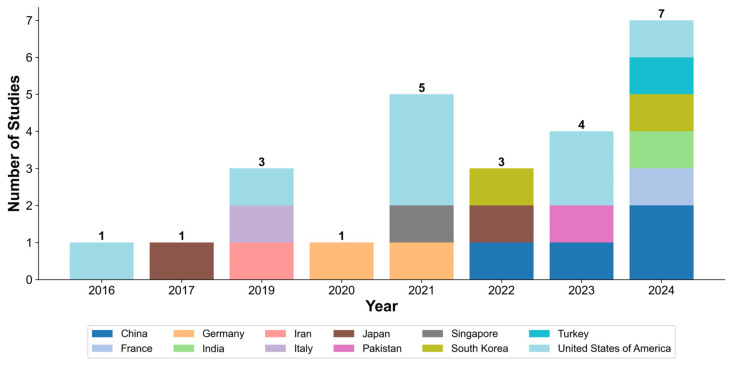
Number of studies in different countries over the years. This graph displays the increasing number of countries participating in research on the application of AI methods for MSCs image analysis, demonstrating a growing international interest in this field. The numbers above each bar indicate the total number of publications for that year.

The number of publications focused on analyzing mesenchymal stem cell images using AI methods has been steadily increasing, with the highest number of publications in 2024 (seven studies) [21,35,38,39,41,46,49]. Over the past decade, there has been a notable increase in the number of countries whose scientific teams are involved in this research area (Figure 3). Table 2. A concise summary of included studies. The full version with detailed descriptions is available in Table S2.

### 3.3. Areas of AI Application in MSC Image Analysis

**Figure 4:**
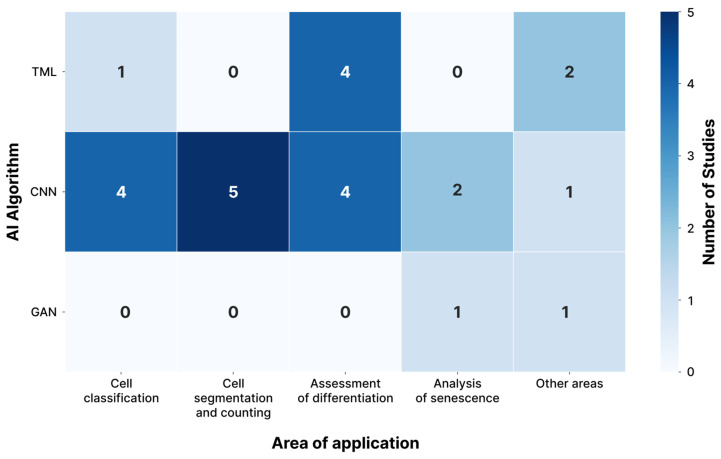
Heatmap of AI algorithm prevalence across application areas. This heatmap illustrates the types of AI algorithms (Y-axis), including traditional machine learning (TML: SVM, K-means, random forest, etc.), convolutional neural networks (CNN), and generative adversarial networks (GAN), and their most frequent usage in different application areas for MSCs image analysis (X-axis), such as cell classification, segmentation, differentiation assessment, and senescence analysis.

This section addresses key areas of AI application for MSCs image analysis identified through the review. Inductive analysis of the 25 included studies, based on the research objectivity, revealed that AI methods are primarily applied to solve the following five tasks: (1) cell classification (20%, *n* = 5) [20,21,39,40,50]; (2) cell segmentation and counting (20%, *n* = 5) [41,43,46,47,51]; (3) assessment of differentiation (32%, *n* = 8) [29,32,34,35,36,37,42,44]; (4) analysis of senescence (12%, *n* = 3) [33,38,49]; (5) other applications (16%, *n* = 4) [30,31,45,48]. Detailed distribution of the applied AI algorithms across specific tasks within each of these areas is clearly illustrated in the heatmap (Figure 4), where CNNs notably emerge as the most frequently utilized algorithm for cell classification, segmentation and counting, and differentiation assessment tasks, demonstrating the predominant usage trends.

#### 3.3.1. Cell Classification

D’Acunto et al. used deep learning to classify MSCs differentiated into osteoblasts (from healthy bone tissue) and osteosarcoma cells [50]. In their work, Kim et al. employed CNNs to classify MSC lines with high and low MUSE marker content based on live cell images [40]. Liu et al. used a hyperspectral imaging-based CNN (H-SCNN) to classify MSCs with high and low functionality [39]. The authors of the study by Mota et al. used traditional machine learning to classify MSCs phenotypes (actively and slowly dividing) based on morphometric features extracted from segmented cells [20]. Mukhopadhyay et al. developed a CNN for binary classifying of stem cells from exfoliated human deciduous teeth (SHED) and Wharton’s jelly MSCs using imaging flow cytometry (IFC) [21].

Analysis of research in this category indicates that AI models, particularly CNNs, achieve high classification accuracy, often exceeding 97% [21,50]. This success is attributed to their ability to automatically extract complex hierarchical features from images, surpassing traditional machine learning approaches that rely on predefined morphological parameters. However, efficacy and applicability depend heavily on the imaging technology employed. While standard bright-field microscopy is the most accessible, specialized techniques such as imaging flow cytometry (Mukhopadhyay et al. [21]) or hyperspectral imaging (Liu et al. [39]) enable high-content analysis and provide richer data, leading to enhanced classification accuracy. Consequently, the choice of the optimal method involves a trade-off between the required accuracy, throughput, and equipment availability in a given laboratory or clinical setting. For model validation, authors primarily utilize metrics such as accuracy, the area under the ROC curve (AUC) and the F1-score.

#### 3.3.2. Cell Segmentation and Counting

Adnan et al. used transfer learning with DeepLab for semantic segmentation of MSCs images [47]. Halima et al. developed an algorithm combining a DAE and a U-Net for cell segmentation and deformability assessment in noisy images [46]. Zhang et al. proposed a CNN for automatic counting of MSCs nuclei in light microscopy images [51]. Ochs et al. developed an algorithm based on the U-Net for automatic cell confluency assessment [43]. Ngo et al. showed that InceptionV3 and ResNet50 effectively determine MSCs confluency in single- and multilayer flasks [41].

The analysis of the included studies reveals the strengths and weaknesses of different architectures. Standard models such as U-Net demonstrate high efficacy in segmentation tasks, as shown by Ochs et al. [43]. However, their performance can degrade on noisy images. Halima et al. address this issue by applying a denoising autoencoder (DAE) before the U-Net, which significantly enhances segmentation accuracy (precision of 81% for DAE + U-Net versus 59% for U-Net alone) [46]. This finding underscores the importance of approaches tailored to the source data quality. The primary validation in these studies are the Dice coefficient and the F1-score, which are standard for evaluating segmentation and counting tasks.

#### 3.3.3. Assessment of Differentiation

Chen et al. used SVM to classify cells based on osteogenic differentiation under poly-(ε-caprolactone) nanofibers, using morphology as input data [29,32]. Tanaka et al. developed an SVM-based algorithm to differentiate adipogenic, osteogenic, and undifferentiated cells in agarose microwells, studying geometric constraint effects on differentiation [44]. Lan et al. applied transfer learning with VGG16, InceptionV3, and ResNet50 for quantitative analysis of MSCs osteogenic differentiation from F-actin/DAPI-stained single cell images [36]. Hoffman et al. created a CNN model to predict early MSCs differentiation based on fluorescent images of the nucleus and actin cytoskeleton [35]. Kong et al. clustered cells into undifferentiated, differentiating, and differentiated categories using the K-means++ algorithm and FLIM-derived morphological parameters [37]. Dursun et al. used VGG16 to classify cell types (MSCs, chondrocytes, and tenocytes) from light microscopy images [42]. Mai et al. employed pre-trained models (VGG19, InceptionV3, ResNet18, ResNet50) to predict MSCs osteogenic and adipogenic differentiation potential [34].

Research in this field highlights a fundamental trade-off between interpretability and performance. Traditional methods, such as SVMs employed by Chen et al. [29,32] and Tanaka et al. [44], offer greater transparency as they are based on well-defined morphological features. In contrast, deep learning approaches, utilized by Lan et al. [36] and Mai et al. [34], consistently demonstrate superior performance (e.g., an AUC of 0.94 in Lan et al. [36]), reflecting their ability to capture subtle morphological shifts beyond predefined feature sets. The choice between these approaches is ultimately task-dependent: interpretability offered by SVMs may be preferable for exploratory research, whereas the accuracy of CNNs is essential for high-throughput screening.

#### 3.3.4. Analysis of Senescence

Celebi et al. developed an algorithm for automatic segmentation and counting of senescent MSCs based on Mask R-CNN and a self-supervised learning [49]. He et al. developed a ResNet algorithm for detecting senescent cells in images [38]. Weber et al. developed a conditional generative adversarial network (cGAN) to predict immunofluorescent images of senescence markers (SABG, p16, p21, and p38) from MSCs phase-contrast images [33].

Research in this area demonstrates a significant paradigm shift towards non-invasive, real-time assessment of cellular senescence–a key advantage over traditional destructive methods such as SA-β-gal staining. The applied AI approaches can be broadly categorized into two groups: detection and segmentation models [38,49] and generative models for “virtual staining” [33]. In the first group, R-CNN-based architectures (Cascade R-CNN, Mask R-CNN) achieve high precision in detecting and counting senescent cells. Notably, Celebi et al. employed self-supervised learning (SSL) to reduce reliance on large annotated datasets, marking a crucial step towards practical implementation [49]. Conversely, Weber et al. demonstrated a highly promising approach using conditional generative adversarial networks (cGANs) to predict molecular senescence markers expression from standard bright-field images [33].

This virtual staining approach preserves cell culture viability for downstream applications, which is critically important for the production of cell-based therapeutics. Validation in these studies correlates AI outputs with the expression of established biological markers (e.g., p16, p21), confirming the biological relevance of the captured morphological features.

#### 3.3.5. Other Areas

Hassanlou et al. used a fully convolutional regression network for automatic lipid droplet counting in adipogenic differentiation assessment [48]. Suyama et al. developed a model predicting MSCs proliferation passage number from cell morphology using linear regression with LASSO regularization and a random forest (RF) algorithm [45]. Marklein et al. employed visual stochastic neighbor embedding (VisNE) with linear discriminant analysis (LDA) to predict MSCs phenotyping across 14 subpopulations based on morphology [30]. Imboden et al., using a conditional generative adversarial network (cGAN), developed a method for converting phase-contrast microscopy images into quantitative measurements of MSC-specific marker expression levels [31].

These studies demonstrate AI’s versatility, extending beyond standard classification and segmentation to highly specialized quantitative assessments. Two principal directions emerge: regression models for quantification [43,46] and generative or exploratory models for identifying complex phenotypes [28,29]. Regression approaches measure parameters such as lipid droplet count or proliferative potential (passage number), thereby translating visual data into quantitative quality metrics. Generative models, exemplified by Imboden et al.’s “virtual staining”, enable for the non-invasive estimation of specific MSCs marker expression levels [29]. Marklein et al. use of viSNE illustrates AI’s role in scientific discovery, identifying novel morphological subpopulations correlated with functional potency [28].

A key strength of these methods is their ability to extract deep functional information from standard micrographs. However, their complexity necessitates rigorous validation using diverse metrics, such as the correlation coefficient, mean absolute error (MAE), and prediction accuracy. Collectively, these approaches transform AI from an automation tool to a powerful platform for quantitative biology and novel knowledge discovery.

### 3.4. Types of AI Algorithms

**Figure 5:**
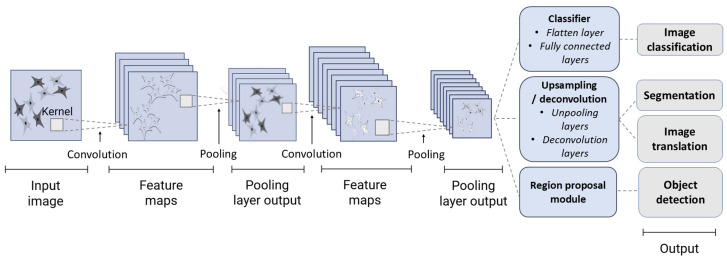
Schematic overview of a convolutional neural network (CNN) architecture and its common applications in MSCs image analysis. The input image is processed through a series of alternating convolutional and pooling layers. Convolutional layers apply kernels to extract hierarchical features (creating feature maps), while pooling layers reduce their spatial dimensions. The resulting high-level feature representation can feed into different modules to perform specific tasks, such as a classifier for image classification, an upsampling or deconvolutional module for pixel-wise tasks like segmentation and image translation, and a region proposal module for object detection.

The heatmap, presented in Figure 4, visually summarizes the prevalent AI algorithm types across various application areas, offering a comprehensive overview of their interconnected usage. Deep learning, particularly convolutional neural networks (CNNs), is the most common AI method for MSCs image analysis (64%, *n* = 16). CNNs analyze biomedical images by extracting spatial features using convolutional computational layers with learnable filters (kernels). Pooling layers reduce dimensionality, ensuring invariance to shifts and scaling. The resulting feature maps represent local patterns (lines, edges, corners, curves, textures, etc.), processed by fully connected layers for classification or regression tasks like pathology detection, cell type classification, or structure segmentation (Figure 5).

Studies used both custom neural networks [21,35,48,51] and state-of-the-art (SoTA) models such as AlexNet [41], DeepLab [47], DenseNet [40], Inception [34,36,41,50], MobileNet [21,35,41,47], ResNet [21,34,36,39,40,41], ShuffleNet [41], U-Net [31,33,43,46], VGG [21,34,36,39,40,42], XceptionNet [40,47]. Region-based CNNs (R-CNN) like Fast R-CNN [50] and Cascade R-CNN [38] were used for cell detection and localization. Transfer learning improved CNN performance, especially with limited training data [21,40,47,49].

Other machine learning methods were less common for classification, clustering, and anomaly detection. Support vector machines (SVM) classified cells based on morphology [29] and studied nanofibril substrate effects [32]. The K-means algorithm clustered cells and analyzed their heterogeneity. In combination with fluorescence lifetime imaging microscopy (FLIM) or stimulated Raman spectroscopy (SRS), it was also employed for classifying differentiation stages [37]. Linear discriminant analysis (LDA), Bayesian linear regression, and principal component analysis (PCA) were used for dimensionality reduction and improving result interpretation [45].

### 3.5. Effectiveness of AI Methods for MSCs Image Analysis

The analysis of the included studies allowed for an overall assessment of the effectiveness of various AI methods for characterizing MSCs in images. Deep learning models, especially CNNs, were the most effective for classification, segmentation, and differentiation prediction. For example, CNNs achieved 97.5% accuracy in binary cell classification [21], and U-Net-based models showed high segmentation accuracy (81%) even in noisy images [46]. Transfer learning significantly improved CNN performance with limited data [40,47]. At the same time, traditional machine learning methods, such as SVM [40] and K-means [37], were effective in specific scenarios: SVM demonstrated 98.2% accuracy in differentiating regions of differentiation, and K-means successfully clustered cells by differentiation stages based on FLIM data.

The observed superiority of CNNs over traditional machine learning methods (e.g., SVMs) in the analysis of microscopic images of MSCs stems from fundamental differences in their feature extraction and pattern recognition capabilities. Traditional approaches depend on predefined morphological descriptors—such as area, perimeter, and shape—whereas CNNs automatically learn hierarchical representations directly from raw image data. This automated feature learning proves particularly advantageous for characterizing MSCs, where diagnostically relevant information encompasses subtle morphological variations–cell elongation, the morphology of cytoplasmic protrusions, and cytoskeletal texture–that standard feature sets cannot adequately capture. CNN architectures processes information through hierarchical layers of increasing complexity, mirroring the multi-scale nature of MSC morphological analysis. Early convolutional layers detect basic cellular boundaries and textural patterns, while deeper layers integrate this information to recognize complex phenotypic signatures associated with various MSC states, including proliferative potential, differentiation capacity, and senescence markers. This hierarchical integration enables CNNs to distinguish morphologically similar yet functionally distinct MSC populations, which traditional approaches may misclassify. For example, studies using SVMs often employ “supercell” analysis (averaging metrics from small cell groups) to address population heterogeneity [29,32,44]. Although this method enhances classifier stability, it remains dependent on the initial, human-engineered feature set, unlike diverse CNN architectures learning directly from images without manual feature engineering.

A key advantage of AI methods over traditional approaches in MSCs analysis is their non-invasiveness, eliminating cell fixation or staining. Traditional methods, such as immunofluorescent staining of differentiation markers (e.g., ALP for osteogenesis or Oil Red O for adipogenesis) or cell fixation with formaldehyde, irreversibly compromise cell integrity, making impossible performing dynamic observations and using cells for therapeutic purposes. For example, Weber et al. [33] used cGAN to predict senescence markers expression (SABG, p16, p21) from phase-contrast images, avoiding destructive staining. Mai et al. [34] demonstrated that deep learning architectures (VGG19, InceptionV3, ResNet18, ResNet50) applied to live cell imaging data predict MSCs osteogenic and adipogenic differentiation with up to 95.7% accuracy. AI methods can also provide more accurate analysis of MSCs compared to traditional approaches: Hassanlou et al. [48] achieved 94% accuracy with automatic lipid droplet counting (compared to 90–95% with manual staining), and Liu et al. [39] reached 0.918 F1-score in classifying cell functionality using H-SCNN (compared to 0.826 with visual assessment).

Thus, the high accuracy and resource efficiency of AI create a foundation for scaling up biomanufacturing and clinical implementation of cell-based products, where speed, standardization, and cost-effectiveness are critical. To visually summarize the effectiveness, reported trade-offs, and common validation approaches of the discussed AI methods, we have compiled the key findings in Table 3.

## 4. Discussion

### 4.1. Principal Findings and Implications of AI in MSC Analysis

The goal of this review was to identify applications of AI-based image processing methods for analyzing mesenchymal stem cells (MSCs). In this context, the integration of AI into MSCs workflows can be conceptually divided into three stages: (1) automating routine laboratory assessments (e.g., cell counting, confluence evaluation), (2) enabling noninvasive predictive characterization (e.g., forecasting differentiation potential or senescence), and (3) facilitating in-depth phenotypic analysis for research purposes. This review systematizes current advances within this framework.

Traditionally, manual analysis of MSCs cultures using light microscopy is common for live cell evaluation. However, this method is limited by subjective interpretation of morphological characteristics, leading to inconsistent results. Detailed study of MSCs characteristics (senescence, differentiation, marker expression) requires cell fixation and antibody staining. While necessary for visualizing cellular components, it irreversibly damages the cells, preventing dynamic observation of their state. This leads to the loss of time-dependent information crucial for understanding MSCs biology.

In contrast, AI methods offer new opportunities for non-invasive, live analysis of MSCs states, detecting and quantifying cell parameters without physical interference. This enables dynamic observation of cell populations and their responses to different stimuli in real time, providing a more comprehensive understanding of MSCs biology and advancing their therapeutic potential [31,33,43].

### 4.2. Key Limitations and Challenges

Despite significant advancements in applying AI methods for MSCs analysis, several key limitations persist, necessitating further investigation. One major obstacle is the heterogeneity of MSCs, which form a diverse population with varied morphological and functional characteristics. This diversity complicates the development of universal models capable of analyzing all cell types within a population [31,52]. To address this issue, it is crucial to account for intra-population variability and develop methods that can accurately model MSCs behavior and characteristics under different conditions [21,29,32].

Another significant limitation is the lack of standardization in AI methods. Currently, there are no unified protocols for collecting, annotating, and processing MSCs images, which hinders the comparison of results across different studies and limits model portability. In the absence of practices and publicly available annotated datasets, creating universal solutions suitable for a wide range of applications becomes challenging [47]. To enhance research quality and reproducibility, standardized methodologies and accessible datasets need to be developed. This challenge is typical for emerging scientific fields until the most effective practices are established.

Superior performance of CNNs entails practical challenges. These models require significantly larger annotated datasets for effective training compared to traditional methods, often creating a data preparation bottleneck. For example, He et al. assembled a database of over 83,000 annotated cells to train a Cascade R-CNN model [38], whereas many other studies rely on only a few hundred or thousand images. Furthermore, CNNs demand substantial computational resources, particularly graphics processing units (GPUs), which can pose infrastructural challenges. The “black box” nature of deep learning models also requires careful consideration, as understanding the biological rationale behind classification decisions remains critically important. Despite these limitations, the demonstrated ability of CNNs to identify morphological patterns that correlate with the functional states of MSCs underscores their promise for the automated quality assessment of stem cells.

A further significant limitation, which is largely overlooked in the analyzed literature, is the potential for bias in AI models. Models trained on cell images acquired from a single laboratory using specific equipment and protocols may demonstrate significantly degraded performance when applied to data from other sources (a phenomenon known as domain shift). Furthermore, if the training dataset is dominated by cells of a particular morphology, the model may develop a bias towards these, consequently overlooking rarer subpopulations. The absence of bias-mitigation strategies in the reviewed articles represents a conspicuous gap and a critical direction for future research to ensure AI models’ reliability and generalizability of AI models in cell biology.

### 4.3. Future Perspectives and Research Directions

Promising future research directions include the development of more complex and interpretable models capable not only of classifying and segmenting cells but also detecting subtle changes in their state. In particular, research focused on identifying phenotypic and senescence markers using CNNs on brightfield images [31,33] appears particularly promising and warrants further development. There is significant potential in integrating multimodal data, such as imaging, genomic, and metabolomic data, which will enable a more comprehensive analysis of MSCs characteristics.

To address limitations of heterogeneity and the lack of standardized datasets, a collaborative and multifaceted approach is required. Actionable solutions include establishing a community-driven consortium for MSCs image annotation to develop standardized labeling protocols and a unified platform for creating large, high-quality public datasets. Additionally, dedicated benchmarking platforms with standardized datasets and evaluation metrics would facilitate reproducible research and objective AI algorithm comparison. To overcome data-sharing barriers while preserving privacy, integrating federated learning approaches is highly recommended, enabling robust model training on decentralized data without direct data exchange.

Concurrently, the future development of tools for automatic annotation will be crucial for improving the accuracy and reproducibility of AI models. The application of AI in automated MSCs culturing systems could optimize manufacturing processes, ensuring improved quality of cell-based products for therapeutic use [43].

Currently, AI regulation in the biomedical field is actively evolving [53,54], with key focus areas including risk management, ethics, transparency, and harmonization of international standards. A primary regulatory challenge is classifying AI systems as “software as a medical device” (SaMD), which necessitates novel validation approaches capable of accounting for the adaptive nature of learning algorithms [55]. The absence of global consensus on regulatory strategies and insufficient transparency in validation processes limits the safe and effective integration of AI into clinical practice. Moreover, serious ethical concerns arise related to the explainability problem (explainable AI, xAI) of AI models [56]. Deep learning algorithms, such as CNNs, often function as “black boxes”, producing decisions that are difficult for humans to interpret. In clinical contexts where AI decisions can affect therapeutic product quality, lack of transparency is unacceptable and creates accountability issues while undermining clinician trust in the technology [57]. Therefore, future research must prioritize developing interpretable AI models that can provide clear rationales for their predictions, enabling clinicians to understand and verify the basis of AI assessments. For instance, rather than simply classifying a cell as “senescent”, an interpretable model could highlight specific morphological features (e.g., flattened shape, specific nuclear texture) that led to this conclusion, linking AI outputs to established biological knowledge. To address these challenges, concepts of “good machine learning practice” (GMLP) are being developed, which include principles for ensuring data quality, transparency, interpretability, and accountability throughout the medical device lifecycle [58,59,60]. Overcoming these issues requires collaborative regulatory strategies, responsible innovation practices involving all stakeholders, and continuous refinement of regulatory frameworks for successful implementation of AI in clinical MSC manufacturing and therapy.

## 5. Conclusions

A systematic analysis of 25 studies published between 2014 and 2024 confirms that AI in MSC image analysis is rapidly advancing. Our findings reveal the clear dominance of CNNs, which are employed in 64% of the reviewed studies for addressing key tasks such as differentiation assessment, cell classification, and segmentation. Achieving accuracies of up to 97.5%, AI models significantly outperform traditional approaches, enabling non-invasive, dynamic, and objective monitoring of live cell cultures–a critical step toward improving the quality of cell-based therapies.

Despite these advances, translating research findings into robust clinical tools faces significant hurdles. Key challenges, such as inherent heterogeneity of MSC populations, lack of standardized protocols for image acquisition and annotation, and limited availability of public datasets, currently impede the generalizability of models and hinder their external validation.

Looking forward, future work must shift from demonstrating accuracy to ensuring reliability, interpretability, and clinical translatability. Addressing the “black box” nature of many deep learning models is essential, as this opacity remains a major barrier to clinical adoption. Developing and applying explainable AI (xAI) techniques will be paramount for regulatory approval and clinician trust. Furthermore, next-generation models should integrate multimodal data–combining visual features with genomic, proteomic, or metabolic profiles–to create a holistic view of cell state. Achieving such interpretable, multimodal systems depends on overcoming data scarcity through collaborative efforts to establish high-quality, open-access annotated image datasets and standardized platforms for objective model comparison.

Ultimately, AI is poised to revolutionize MSC biomanufacturing and therapy by transforming a subjective, manual process into a standardized, automated, and predictive science. Addressing current challenges through focused efforts to create interpretable models, integrate multimodal data, and build collaborative research infrastructure will be essential for unlocking AI’s full potential in regenerative medicine.

## Figures and Tables

**Table 1:** Inclusion and exclusion criteria for studies.

Inclusion Criteria	Exclusion Criteria
Studies on MSCs from animals and humans	Studies involving objects other than MSCs
Use of AI methods for MSCs image analysis	Use of AI methods for purposes other than image analysis
Studies published in the last 10 years	No use of AI methods
Access to full text	Reviews
	Preprints and conference abstracts
	Unavailable full text

**Table 2:** A concise summary of included studies. The full version with detailed descriptions is available in Table S2.

Authors, Year, Country	Study Objective	Cell Type and Origin	AI Algorithm	Dataset Description	Research Outcomes	Ref.
Chen et al., 2016, USA	Classification of cell morphology on PCL substrates	Human bone marrow MSCs	SVM	Microscopy images of cells on PCL substrates	Identified key morphological indicators; supercell (group of cells) analysis improved accuracy	[29]
Tanaka et al., 2017, Japan	Differentiation analysis in agarose microwells	Commercial human bone marrow MSCs	SVM	Annotated microscopy images of cell regions	Achieved 98.2% pixel-level classification accuracy	[44]
Marklein et al., 2019, USA	Identification of MSC subpopulations post-IFN-γ stimulation	Commercial bone marrow MSCs	viSNE with LDA	Phase-contrast images of manually segmented cells	Identified subpopulations correlated with T-cell inhibition	[30]
Hassanlou et al., 2019, Iran	Automated counting of lipid droplets	Differentiated mouse bone marrow MSCs	Fully convolutional regression network	Cropped microscopy images	Achieved 94% counting accuracy, outperforming manual methods	[48]
D’Acunto et al., 2019, Italy	Classification of osteosarcoma vs. MSCs	Human bone marrow MSCs and MG-63 cells	Faster R-CNN with Inception ResNet v2	Augmented microscopy images of 5 cell classes	Achieved up to 97.5% classification accuracy	[50]
Dursun et al., 2020, Germany	Recognition of tenogenic differentiation	Differentiated bone marrow MSCs	VGG16-based CNN	Augmented light microscopy images	Model accuracy of 92.2%	[42]
Mota et al., 2021, USA	Segmentation and classification of cell replication speed	Human bone marrow MSCs	Custom algorithm with LSVM, LDA, etc.	Phase-contrast images of segmented cells	Effective for low/mid-density cultures (AUC up to 0.816)	[20]
Zhang et al., 2021, Singapore	Detection of cell nuclei in brightfield images	Commercial human MSCs	CNN ensemble	Brightfield images of fixed and live cells	Achieved F1-score of 0.985 on fixed cells	[51]
Imboden et al., 2021, USA	Quantitative prediction of marker expression from phase-contrast images	Commercial human bone marrow MSCs	cGAN with U-Net	Paired phase-contrast and immunofluorescence images	Enabled label-free tracking of protein distribution (Corr. Coeff. 0.77)	[31]
Ochs et al., 2021, Germany	Automated confluency assessment for quality control	Human adipose tissue MSCs	U-Net	Augmented microscopy images	Achieved F1-score of 0.833 in a high-throughput system	[43]
Chen et al., 2021, USA	Prediction of osteogenic differentiation based on morphology	Human bone marrow MSCs	SVM	Synthetic datasets from morphometric data	Correlated morphology with osteogenic potential	[32]
Lan et al., 2022, China	Quantitative assessment of osteogenic differentiation	Rat bone marrow MSCs	InceptionV3, VGG16, ResNet50	Confocal images of stained cells	InceptionV3 achieved AUC of 0.94, outperforming SVM	[36]
Suyama et al., 2022, Japan	Noninvasive prediction of high-potency MSC subpopulations	Human bone marrow MSCs	LASSO regression and RF	Time-series morphological data	Predicted cell potency from morphological data; RF/LASSO outperformed	[45]
Kim et. al., 2022, South Korea	Identification of MUSE cells based on differentiation potential	Human nasal turbinate-derived MSCs	Transfer learning (DenseNet121, etc.)	Brightfield images validated via immunofluorescence and flow cytometry	DenseNet121 achieved highest AUC (0.975) and accuracy (92.2%)	[40]
Weber et al., 2023, USA	Prediction of senescence markers from phase-contrast images	Commercial human adipose and bone marrow MSCs	U-Net-based cGAN	Paired phase-contrast/immunofluorescence images	Strong correlation between predicted and actual senescence markers	[33]
Kong et al., 2023, China	Differentiation analysis using FLIM and SRS imaging	Human MSCs	K-means++ clustering on FLIM/SRS data	Single-cell FLIM/SRS images	Successfully tracked differentiation stages; validated by staining	[37]
Adnan et al., 2023, Pakistan	Semantic segmentation of MSCs	Commercial human bone marrow MSCs	DeepLab variants	EVICAN dataset (blurred and normal images)	Achieved >99% accuracy; one variant showed better generalizability	[47]
Mai et al., 2023, USA	Prediction of differentiation potential from live cell imaging	Human bone marrow MSCs	VGG19, InceptionV3, ResNet18/50	Time-series images of differentiating cells	ResNet50 achieved >95% accuracy and AUC >0.99	[34]
He et al., 2024, China	Detection of senescent cells	Induced pluripotent stem cell-derived MSCs	Cascade R-CNN with ResNet	Annotated images of SA-β-gal-stained cells	Achieved mAP of 0.81; correlated with senescence markers	[38]
Celebi et. al., 2024, Turkey	Segmentation of senescent cells	Commercial human adipose tissue MSCs	Mask R-CNN with SimCLR-based SSL	Images for self-supervised learning and fine-tuning	SSL improved mAP by 8.3%; outperformed U-Net and DeepLabV3	[49]
Mukhopadhyay et al., 2024, India	Classification of SHED vs. HWJ MSCs via imaging flow cytometry	SHED and HWJ MSCs	Custom CNNs and transfer learning	Single-cell brightfield images	Achieved 97.5% accuracy	[21]
Halima et al., 2024, France	Cell segmentation and deformability assessment	Human adipose tissue MSCs	Autoencoders (DAE/VAE) and U-Net	Microfluidic images	DAE + U-Net achieved highest precision (81%)	[46]
Liu, 2024, China	Functional classification of MSCs via hyperspectral imaging	Commercial human bone marrow MSCs	Hyperspectral separable CNN (H-SCNN)	Hyperspectral images annotated by flow cytometry	H-SCNN achieved 89.6% accuracy, outperforming ResNet/VGG	[39]
Hoffman et al., 2024, USA	Determination of stemness and early differentiation	Commercial human bone marrow MSCs	Custom CNN vs. MobileNet	Time-series fluorescent images of actin/chromatin	Achieved up to 90% accuracy with combined actin/chromatin images	[35]
Ngo et al., 2024, South Korea	Confluency assessment and anomaly detection	Human Wharton’s jelly MSCs	Ensemble of CNNs and Vision Transformer	Monolayer and multilayer flask images	High accuracy for confluency (AUC 0.958) and anomaly detection	[41]

**Table 3:** A comparative summary of AI approaches in MSC image analysis.

Application Area	Primary Methods	Reported Strengths	Reported Weaknesses/Trade-Offs	Typical Validation Metrics
Cell classification	CNN, SVM	CNN: high accuracy, automatic feature extraction. SVM: high interpretability.	CNN: “black-box” nature, requires large datasets. SVM: requires manual feature engineering.	Accuracy, AUC, F1-score
Segmentation and counting	U-Net, DeepLab, DAE	U-Net: high precision on clean images. DAE + U-Net: robustness to image noise.	High dependency on large, pixel-level annotated datasets.	Dice coefficient, F1-score, precision, IoU
Differentiation assessment	CNN, SVM, k-means	CNN: enables non-invasive prediction on live cells. SVM/k-means: transparent, based on defined features.	SVM/k-means: lower accuracy with subtle morphological changes.	AUC, correlation with biochemical assays
Senescence analysis	cGAN, Mask R-CNN	cGAN: “virtual staining” preserves cell viability. R-CNN: Precise detection and segmentation.	Computationally intensive, complex to train, require large datasets.	Correlation with senescence markers, mAP

## Data Availability

The original contributions presented in this study are included in the article. Further inquiries can be directed to the corresponding authors.

## Supplementary Materials

The following supporting information can be downloaded at https://www.mdpi.com/article/10.3390/jimaging11100371/s1: Table S1: Searching strategy. Table S2: Data extracted from included studies.

## Abbreviations

The following abbreviations are used in this manuscript:

AI	Artificial Intelligence
AUC	Area Under the Curve
cGAN	Conditional Generative Adversarial Network
DAE	Denoising Autoencoder
FLIM	Fluorescence Lifetime Imaging Microscopy
H-SCNN	Hyperspectral Separable Convolutional Neural Network
HWJ	Human Wharton’s Jelly
IFN-γ	Interferon-gamma
IoU	Intersection over Union
LASSO	Least Absolute Shrinkage and Selection Operator
LDA	Linear Discriminant Analysis
LSVM	Linear Support Vector Machine
mAP	Mean Average Precision
MRI	Magnetic Resonance Imaging
MSCs	Mesenchymal Stem/Stromal Cells
PCL	Polycaprolactone
R-CNN	Region-Based Convolutional Neural Network
RF	Random Forest
ROC	Receiver Operating Characteristic
SHED	Stem Cells from Human Exfoliated Deciduous Teeth
SRS	Stimulated Raman Scattering Microscopy
SSL	Self-Supervised Learning
SVM	Support Vector Machine
VAE	Variational Autoencoder
viSNE	Visual Stochastic Neighbor Embedding
