# Supplementary material for: Current Trends and Future Opportunities of AI-Based Analysis in Mesenchymal Stem Cell Imaging: A Scoping Review
Source: J Imaging. 2025 Oct 18;11(10):371. doi: 10.3390/jimaging11100371 (PMC12564989; doi:10.3390/jimaging11100371)
Supplement: Supplementary file 1 [file jimaging-11-00371-s001.zip › S1_Searching strategy.pdf]

Table S1. Searching strategy:

Current trends and future opportunities of AI-based analysis in mesenchymal stem cell imaging: a scoping review

Searched databases: PubMed, Dimensions.AI, Cochrane (Wiley), Scopus, Elibrary, Google Scholar
Searching Date: 13/10/2024
Last 10 years.

PubMed:

Search	Query	Field	Records retrieved
#1	"AI" OR "artificial intelligence" OR "convolutional neural network" OR "deep learning" OR "machine learning" OR "transfer learning"	Title and abstr act	13,363
#2	"mesenchymal stem cell*" OR "MSC" OR "MSCs" OR "hMSC*" OR "BMSC*" OR "hBMSC*"	Title and abstr act	59,028
#3	"authenticating" OR "authentication" OR "classification" OR "detecting" OR "detection" OR "identification" OR "image processing" OR "imaging" OR "predicting" OR "prediction" OR "screening"	Title and abstr act	2,369,090
#4	#1 AND #2 AND #3		145

Dimensions:

Search	Query	Field	Records retrieve d
#1	"AI" OR "artificial intelligence" OR "convolutional neural network" OR "deep learning" OR "machine learning" OR "transfer learning"	Title and abstract	839,195
#2	"mesenchymal stem cell*" OR "MSC" OR "MSCs" OR "hMSC*" OR "BMSC*" OR "hBMSC*"	Title and abstract	89,842
#3	"authenticating" OR "authentication" OR "classification" OR "detecting" OR "detection" OR "identification" OR "image processing" OR "imaging" OR "predicting" OR "prediction" OR "screening"	Title and abstract	5,510,37 2
#4	#1 AND #2 AND #3		306

Scopus:

Search	Query	Field	Records retrieved

#1	TITLE-ABS-KEY("AI" OR "artificial intelligence" OR "convolutional neural network" OR "deep learning" OR "machine learning" OR "transfer learning")	Article title, Abstract, Keywords	-
#2	TITLE-ABS-KEY("mesenchymal stem cell*" OR "MSC" OR "MSCs" OR "hMSC*" OR "BMSC*" OR "hBMSC*")	Article title, Abstract, Keywords	-
#3	TITLE-ABS-KEY("authenticating" OR "authentication" OR "classification" OR "detecting" OR "detection" OR "identification" OR "image processing" OR "imaging" OR "predicting" OR "prediction" OR "screening")	Article title, Abstract, Keywords	-
#3	#1 AND #2 AND #3		440

Cochrane (Wiley):

Search	Query	Field	Records retrieved
#1	AI OR artificial intelligence OR convolutional neural network OR deep learning OR machine learning OR transfer learning	Title/ Abstract/ Keywords	13,363
#2	mesenchymal NEXT stem NEXT cell* OR MSC OR MSCs OR hMSC* OR BMSC* OR hBMSC*	Title/ Abstract/ Keywords	2,935
#3	authenticating OR authentication OR classification OR detecting OR detection OR identification OR image processing OR imaging OR predicting OR prediction OR screening	Title/ Abstract/ Keywords	426,588
#3	#1 AND #2 AND #3		12

Elibrary:

Search	Query	Field	Records retrieved
#1	"AI" OR "artificial intelligence" OR "convolutional neural network" OR "deep learning" OR "machine learning" OR "transfer learning"	Title/ Abstract/ Keywords	224,346
#2	"mesenchymal stem cell*" OR "MSC" OR "MSCs" OR "hMSC*" OR "BMSC*" OR "hBMSC*"	Title/ Abstract/ Keywords	39,595

#3	"authenticating" OR "authentication" OR "classification" OR "detecting" OR "detection" OR "identification" OR "image processing" OR "imaging" OR "predicting" OR "prediction" OR "screening"	Title/ Abstract/ Keywords	182,560
#3	#1 AND #2 AND #3		156

Google Scholar:

Search	Query	Field	Records retrieved
#1	imaging AI, OR artificial OR intelligence, OR convolutional OR neural OR network, OR deep OR learning, OR machine OR learning, OR transfer OR learning "mesenchymal stem cell*"	Anywhere in the article	44
