# Supplementary material for: Current Trends and Future Opportunities of AI-Based Analysis in Mesenchymal Stem Cell Imaging: A Scoping Review
Source: J Imaging. 2025 Oct 18;11(10):371. doi: 10.3390/jimaging11100371 (PMC12564989; doi:10.3390/jimaging11100371)
Supplement: Supplementary file 1 [file jimaging-11-00371-s001.zip › S2_Extracted data.pdf]

Table S2. Data extracted from included studies.

Authors, year, country	Study objective	Cell type and origin	AI Algorithm	Dataset description	Research outcomes	Ref.
Chen et al., 2016, USA.	Development of multidimensional classifiers for analyzing cellular morphological phenotypes on PCL fibrils and films; studying the relationship between cell morphology and osteogenic differentiation.	Human bone marrow MSCs.	Support vector machine (SVM) for cell classification based on morphological features, including analysis of single cells and "supercells" (group of multiple cells).	Images of 121 cells on PCL fibrils and 114 cells on PCL films. 22-dimensional vectors of morphological features divided into 3 groups: cell size, global shape pattern, local feature.	Combination of minor axis length, compactness, and mean negative curvature were key indicators of cell response to fibrous substrates. Supercell size (≥57 cells grouped into clusters of size 4) improved classification accuracy and stability.	[29]
Tanaka et al., 2017, Japan.	Investigation of geometric constraints on cell differentiation in agarose microwells.	Commercial human bone marrow MSCs (Poietics).	SVM for classifying adipogenic, osteogenic, and undifferentiated regions using color images.	Training dataset: 17 small images with annotated regions of adipogenic, osteogenic, and undifferentiated cells.	Pixel-level classification accuracy: 98.2%. Cells in the center of microwells differentiated into adipocytes, while peripheral cells became osteocytes. Undifferentiated cells persisted in peripheral regions even in differentiation-promoting media.	[44]
Marklein et al., 2019, USA.	Identification of morphological subpopulations in MSCs after interferon-gamma (IFN-γ) stimulation.	Commercial bone marrow MSCs from 11 donors (Lonza, AllCells).	Visual stochastic neighbor embedding (viSNE) combined with linear discriminant analysis (LDA) for subpopulation prediction.	1,011 manually segmented cells from 50 phase-contrast images (CellProfiler software). 21-dimensional morphological vectors.	Identified 14 subpopulations correlated with MSCs ability to inhibit CD4+/CD8+ T-cell activation, critical for immune response modulation.	[30]
Hassanlou et al., 2019, Iran.	Algorithm for intracellular image processing to count lipid droplets in differentiated cells.	Mouse femoral bone marrow MSCs differentiated into adipocytes.	Fully convolutional regression network: convolutional component for feature extraction, regression component for lipid droplet counting.	200 images (300×300 pixels) cropped from 1200×1600 microscopy images. Pixel-wise annotations of lipid droplets.	Achieved 94% average counting accuracy, outperforming traditional methods like Oil Red O staining and manual counting.	[48]
D'Acunto et al., 2019, Italy.	Classification of osteosarcoma and MSCs using deep learning.	Human bone marrow MSCs and osteosarcoma cell line MG-63.	Faster R-CNN with Inception ResNet v2 for object detection and feature extraction.	60 images (279 objects) across 5 classes: single cancer cell, cancer cluster, single mesenchymal cell, mesenchymal cluster,	RGB image classification accuracy: 97.5%; grayscale: 97.2%.	[50]

				artifact. Augmented to 480 images.		
Dursun et al., 2020, Germany.	Recognition of tenogenic differentiation in MSCs based on morphology.	Bone marrow MSCs differentiated into tenocytes and chondrocytes (negative control group).	VGG16-based CNN for classifying chondrocytes, MSCs, and tenocytes.	7,500 training and 1,500 test images (1280×960 pixels, 10× magnification) resized to 64×64 pixels with augmentation.	Model accuracy: 92.2% in identifying tenogenic differentiation.	[42]
Mota et al., 2021, USA.	Segmentation and classification of rapidly (RS) and slowly replicating (SR) cells based on morphology.	Human bone marrow MSCs.	Custom algorithm extracting morphometric/textural features with next classifiers: LSVM, RSVM, LDA, KNN, LR.	Cells extracted from phase-contrast images. Training: 472 cells (307 RS, 165 SR). Validation: 186 cells (121 RS, 65 SR).	Sørensen–Dice coefficient: 0.849. AUC: 0.816 (early) and 0.787 (mid-log phase). Sensitivity: 88%; precision: 86%. Effective for low/mid-density cultures but less reliable for high-density cells.	[20]
Zhang et al., 2021, Singapore.	Deep learning-based detection of cell nuclei in brightfield images under varying defocus levels.	Commercial human MSCs (Lonza).	10 CNNs with different architectures trained to predict nuclei pixels. Custom algorithm for nuclei-reference matching.	Three datasets: MID1 (training: 78–80 images), MID2/MID3 (testing: 30–42 images). Fixed/stained and live cells.	Best model achieved F1-scores of 0.985 (fixed/stained) and ~15% lower accuracy for live cells. Performance optimized when training and test samples had similar optical properties.	[51]
Imboden et al., 2021, USA.	Transformation of phase-contrast microscopy images into quantitative measurements of marker expression (CD105, CD90, etc.).	Commercial human bone marrow MSCs (ATCC).	Conditional generative adversarial network (cGAN) with U-Net for image translation.	600–1,500 paired phase-contrast and immunofluorescence images (380×380 μm field of view).	Correlation coefficient: 0.77 for eight markers. Enabled spatial-temporal tracking of protein distribution and heterogeneity.	[31]
Ochs et al., 2021, Germany.	Automated robotic laboratory with deep learning-based quality control via confluency assessment.	Human adipose tissue MSCs.	U-Net with batch normalization for cell detection and confluency estimation.	19 images split into 56,700 augmented patches. Tested on 2,187 fragments.	F1-score: 0.833. System processed >95 culture plates/day.	[43]
Chen et al., 2021, United States of America.	Early morphology-based assessment of osteogenic differentiation modulated	Human bone marrow MSCs from two donors.	SVM classifier trained on 12 2D and 29 3D morphometric metrics from "supercells" (5-cell groups).	100 synthetic datasets generated from morphometric data of	High nanofiber density correlated with increased osteogenic potential. At least two metrics	[32]

	by poly(ε-caprolactone) nanofiber density.			undifferentiated/different iated cells.	(including one 3D) recommended for reliable prediction.	
Lan et al., 2022, China.	Deep learning algorithm for quantitative assessment of osteogenic differentiation.	Rat bone marrow MSCs.	InceptionV3, VGG16, ResNet50 pre-trained on ImageNet and fine-tuned.	2,916 confocal images (F-actin/DAPI-stained cells) across 0, 1, 4, and 7 days of differentiation.	InceptionV3 achieved AUC 0.94 ± 0.04, outperforming SVM and traditional morphology analysis. Predictions correlated with biochemical markers.	[36]
Suyama et al., 2022, Japan.	Noninvasiveearly prediction of high-potency MSCs subpopulations via morphological profiling.	CD271+/CD90+ MSCs isolated from human bone marrow mononuclear cells.	LASSO regression and random forest (RF) to predict proliferative potential.	360 morphological parameters collected over 15 time points (6–90 hours).	Potency (passages 7–13) predicted using 24 morphological descriptors. RF/LASSO outperformed single-descriptor analysis.	[45]
Kim et. al., 2022, South Korea.	CNN-based identification of MUSE cells with high/low multilineage differentiation and stress resistance markers.	Human nasal turbinate-derived MSCs	Transfer learning with DenseNet121, VGG19, ResNet50v2, InceptionV3, Xception.	6,120 brightfield images (MUSE-high vs. MUSE-low) validated via immunofluorescence and flow cytometry.	DenseNet121 achieved highest AUC (0.975), accuracy (92.2%), and sensitivity (90.5%).	[40]
Weber et al., 2023, USA.	Prediction of senescence markers (SA-β-gal, p16, p21, p38) from phase-contrast images.	Commercial immortalized human adipose tissue MSCs (ATCC) and bone marrow MSCs (Lonza).	U-Net-based cGAN trained on paired phase-contrast/immunofluorescence images.	~600 images per channel (phase-contrast, 405 nm, 488 nm, 597 nm; 0.38×0.38 mm field of view).	Strong correlation between predicted and actual senescence marker expression in stress-induced and replicative senescence models.	[33]
Kong et al., 2023, China.	Machine learning model for differentiation analysis using FLIM and SRS imaging.	MSCs from Cell Bank of the Chinese Academy of Sciences.	K-means++ clustering based on NAD(P)H fluorescence lifetime, lipid content, and morphology.	150–200 single cell images per differentiation stage (days 1–28).	Model sensitivity increased over time, showing reduced undifferentiated and increased differentiated cells. Results validated via staining.	[37]
Adnan et al., 2023, Pakistan.	Transfer learning-based semantic segmentation of cells in microphotographs.	Commercial human bone marrow MSCs (Lonza).	DeepLab variants: Algorithm 1 (MobileNet-v2, blurred background), Algorithm 2 (XceptionNet, normal background).	EVICAN dataset: 139 (blurred) and 37 (normal) images.	Algorithm 1: 99.2% accuracy, Dice 99.7%, IoU 0.84. Algorithm 2: 96.3% accuracy, Dice 98.4%, IoU 0.48. Algorithm 2 showed better clinical generalizability.	[47]

Mai et al., 2023, USA.	Prediction of osteogenic/adipogenic differentiation using live cell imaging and deep learning.	Human bone marrow MSCs from 4 donors (Lonza, PromoCell).	VGG19, InceptionV3, ResNet18, ResNet50 for classification.	2,336 images (2592×1944 pixels) across control, adipogenic, osteogenic, and mixed groups at days 1, 2, 3, 5, 7, 10, 13.	ResNet50 achieved highest accuracy: 95.7% (binary) and 94.7% (multiclass). AUC: 0.9958/0.9836. Accuracy dropped to ~88% on days 3/5 due to heterogeneity.	[34]
He et al., 2024, China.	Detection of senescent cells via morphological features.	Induced pluripotent stem cell-derived MSCs.	Cascade R-CNN with ResNet, FPN, RPN, and GN modules.	2,382 training, 797 validation, and 807 test images (640×640 pixels; SA-β-gal-stained). 56,115 senescent and 27,550 normal cells annotated.	mAP: 0.81; recall: 0.93. Senescent cell proportion correlated with senescence markers (p16, p21, IL-6, etc.) and inversely with stemness genes (NANOG, SOX2).	[38]
Celebi et. al., 2024, Turkey.	Segmentation and counting of senescent cells using SSL-enhanced Mask R-CNN.	Commercial human adipose tissue MSCs (ATCC).	Mask R-CNN with SimCLR-based SSL and MSCOCO transfer learning.	342 images (2592×1944 pixels). 8,719 patches for SSL; 100 fully annotated images for fine-tuning.	SSL improved mAP by 8.3% and mAR by 4.1%. Final mAP: 0.736; mAR: 0.819. Outperformed U-Net and DeepLabV3.	[49]
Mukhopadhyay et al., 2024, India.	Binary classification of SHED (human exfoliated deciduous teeth) and Human Wharton's jelly MSCs populations via imaging flow cytometry.	SHED and HWJ MSCs.	Custom CNNs (A: 13 layers; B: regularized first layer) and transfer learning (VGG16, ResNet50, MobileNet).	91,574 brightfield images (180×180 pixels) of single cells.	Model B achieved 97.5% accuracy. Significant morphological heterogeneity within and between SHED and HWJ MSCs populations.	[21]
Halima et al., 2024, France.	Cell segmentation and deformability assessment using supervised/unsupervised learning.	Human adipose tissue MSCs.	Autoencoders DAE/VAE for denoising and U-Net for segmentation.	480 microfluidic images (120 for testing).	DAE + U-Net achieved 81% precision vs. 78% (VAE + U-Net) and 59% (U-Net alone).	[46]
Liu, 2024, China.	Classification of MSCs functionality via hyperspectral imaging.	Commercial human bone marrow MSCs (Guangzhou GeniBio Biotechnology).	Hyperspectral imaging-based separable CNN (H-SCNN) compared to ResNet/VGG.	1,400 grayscale images (220×330 pixels) annotated via flow cytometry (CD73/CD90 expression).	H-SCNN achieved 89.6% accuracy (F1: 0.918) in 21 minutes, outperforming ResNet (36 min) and VGG (35 min). Manual F1: 0.826.	[39]
Hoffman et al., 2024, USA.	Microscopy-based determination of stemness and early differentiation.	Commercial human bone marrow MSCs (RoosterBio).	Four-layer CNN (regression and classification outputs) vs. MobileNet.	Fluorescent images of actin/ chromatin at 0, 6, 24, 48, 72 h (403–462 cells per timepoint). Augmented with rotations.	Chromatin-only images: 88% accuracy; chromatin + actin: 90%. Brightfield: 58% (shallow CNN) vs. 64% (MobileNet). Actin-only: 52%.	[35]

Ngo et al., 2024, South Korea.	Classification of confluency levels and abnormal cell detection in microphotographs.	Human Wharton’s jelly MSCs.	Six CNNs (AlexNet, InceptionV3, ResNet50, ShuffleNetV2, MobileNetV3, Vision Transformer) and RetinaNet (ResNet50) for anomaly detection.	720 monolayer and 413 multilayer flask images annotated for confluency (4 levels) and abnormalities.	InceptionV3: ROC-AUC 0.958, PR AUC 0.923, accuracy 0.928 (monolayer). ResNet50: ROC-AUC 0.958, accuracy 0.875 (multilayer). RetinaNet detected anomalies with 0.954 hit rate and 0 false positives.	[41]
